# Nurse-Performed Ultrasound-Guided Peripherally Inserted Central Catheter (PICC) Placement During General Anesthesia: A Retrospective Cohort Study

**DOI:** 10.7759/cureus.104786

**Published:** 2026-03-06

**Authors:** Ryo Koda, Sayo Kondo, Akihito Kakinuma, Takahiko Akahori

**Affiliations:** 1 Nursing/Anesthesiology, Nagoya Tokushukai General Hospital, Tokushukai Medical Corporation, Aichi, JPN; 2 Nursing, Nagoya Tokushukai General Hospital, Tokushukai Medical Corporation, Aichi, JPN; 3 Department of Anesthesia and Critical Care, Teikyo University School of Medicine, Tokyo, JPN; 4 Anesthesiology and Critical Care, Nagoya Tokushukai General Hospital, Tokushukai Medical Corporation, Aichi, JPN

**Keywords:** central venous catheter, gastrointestinal surgery, general anesthesia, nurse practitioner, perioperative care, peripherally inserted central catheter, task sharing, ultrasound-guided vascular access

## Abstract

Background: Central venous access is essential in perioperative management of major abdominal surgery. While centrally inserted central venous catheters (CVCs) are widely used, insertion-related mechanical complications remain a concern. Peripherally inserted central catheters (PICCs) may represent an alternative strategy, particularly when integrated into nurse-led task-sharing workflows. This study evaluated the feasibility and procedural outcomes of ultrasound-guided PICC placement during general anesthesia.

Methods: This single-center retrospective observational study included 86 adult patients undergoing gastrointestinal surgery under general anesthesia who required central venous access between January 2023 and December 2025. Patients were divided into a PICC group (n = 41) and a CVC group (n = 45). The primary outcome was catheter placement time (minutes). Secondary outcomes included catheter dwell time (days), catheter-related bloodstream infection (CRBSI), and catheter occlusion with intraluminal thrombus. CRBSI was assessed in accordance with CDC guidance, and patients were followed until catheter removal.

Results: Baseline characteristics were comparable between groups. Catheter placement time was significantly shorter in the PICC group than in the CVC group (5.5 ± 3.1 vs. 7.0 ± 2.3 minutes, p = 0.013). Catheter dwell time was longer in the PICC group (7.1 ± 3.1 vs. 3.3 ± 1.7 days, p < 0.001). CRBSI incidence did not significantly differ between groups (2.4% vs. 8.9%, p = 0.363). Catheter occlusion with intraluminal thrombus occurred in one patient (2.4%) in the PICC group and in none in the CVC group. All catheter placements were successful (100% in both groups).

Conclusions: Ultrasound-guided PICC placement during general anesthesia was associated with shorter catheter placement time and longer catheter duration than CVC placement in this retrospective cohort. Catheter-related complications were infrequent; however, definitive conclusions regarding comparative safety are limited by the observational design and low event counts. These findings support the feasibility of perioperative PICC use and suggest potential workflow implications within structured nurse-led task-sharing models. Further prospective multicenter studies incorporating direct operating room workflow metrics and catheter-day-based outcomes are warranted.

## Introduction

Central venous access is an essential component of perioperative management in major abdominal surgery. Conventionally, central venous catheters (CVCs) via the internal jugular, subclavian, or femoral veins have been widely used to facilitate hemodynamic monitoring, administration of vasoactive agents, and rapid fluid infusion [1]. In major gastrointestinal and hepatobiliary surgery, maintaining reliable central access is increasingly emphasized within Enhanced Recovery After Surgery (ERAS) protocols to optimize fluid management [2]. However, CVC placement is associated with mechanical complications such as arterial puncture, pneumothorax, and bleeding, particularly in the peri-induction period when patients are hemodynamically vulnerable [3,4].

Peripherally inserted central catheters (PICCs) have been increasingly adopted as an alternative central venous access device in various clinical settings due to their relatively lower risk of insertion-related mechanical complications [5]. The safety and success rates of PICCs have been further enhanced by the standardization of ultrasound-guided techniques, which are now considered a global gold standard for vascular access [6]. While PICCs are commonly used for long-term intravenous therapy, their role in the intraoperative or perioperative setting remains less clearly defined. Recent studies have begun to explore the feasibility of PICC placement during general anesthesia, suggesting its potential to bridge the gap between intraoperative needs and postoperative continuity of care [7].

In parallel with evolving vascular access strategies, healthcare systems worldwide are promoting task sharing and interdisciplinary collaboration to optimize perioperative workflow and resource allocation [8]. In Japan, nurse-led advanced procedural practice has expanded in recent years under structured training programs [9]. This expansion reflects a broader international trend where advanced practice nurses and nurse practitioners (NPs) take on complex procedural roles to improve healthcare delivery [10]. The implementation of nurse-performed PICC placement during general anesthesia may represent a complementary strategy that allows anesthesiologists to focus on advanced airway and hemodynamic management while maintaining procedural safety.

## Materials and methods

Study design

This was a single-center retrospective observational study conducted at a tertiary care hospital in Japan. The study evaluated adult patients who underwent major gastrointestinal surgery under general anesthesia and required central venous access.

Study population

Patients who received either a PICC or a CVC during the perioperative period were included. The study period was from January 2023 to December 2025.

Exclusion criteria included patients with incomplete medical records or those who underwent emergency surgery requiring immediate vascular access before anesthesia induction. In the IRB application, the estimated sample size was approximately 80 cases (about 40 per group). However, as this was a retrospective observational study including all consecutive eligible patients during the predefined study period, the final analyzed cohort consisted of 86 patients (41 in the PICC group and 45 in the CVC group).

Catheter placement procedure

Indications for central venous access at our institution include prolonged operations, higher-acuity patients (ASA-PS ≥ 3), and high-complexity procedures. Device selection (PICC vs CVC) followed an operational workflow rather than randomization. When NP support was available during weekday daytime hours, the NP (who had completed a nationally certified advanced procedural training program in Japan) performed ultrasound-guided PICC placement during general anesthesia. During on-call hours or when the NP was unavailable, an attending anesthesiologist performed ultrasound-guided CVC placement. CVCs were preferentially inserted via the internal jugular vein, with femoral access used when the internal jugular approach was contraindicated or technically difficult. Anesthesiologist-performed PICC placement and NP-performed CVC placement were not performed in this cohort.

Catheter specifications and tip confirmation

PICCs were 6 Fr triple-lumen catheters. All CVCs were 7 Fr triple-lumen catheters. To enable comparable perioperative use (e.g., vasoactive drug administration and central venous pressure monitoring), triple-lumen devices were selected in both groups. Catheter tip position was confirmed by postoperative chest radiography, and no malposition events were identified in this cohort.

Outcome measures

The primary outcome was catheter placement time (minutes). Secondary outcomes included catheter dwell time (days), catheter-related bloodstream infection (CRBSI), and catheter occlusion with intraluminal thrombus. CRBSI was assessed in accordance with CDC guidance, and patients were followed until catheter removal. Catheter occlusion with intraluminal thrombus was defined as catheter dysfunction/occlusion requiring removal, with visible thrombus identified within the catheter lumen upon removal. Routine imaging screening for venous thrombosis was not performed. We also assessed perioperative hemodynamic stability and total operating room time when available.

Statistical analysis

Statistical analyses were performed using EZR (Easy R), version 1.70 (Saitama Medical Center, Jichi Medical University, Saitama, Japan), which is a graphical user interface for R (The R Foundation for Statistical Computing, Vienna, Austria). Continuous variables were expressed as mean ± standard deviation and compared between groups using the Student’s t-test (Welch’s correction when appropriate). Categorical variables were analyzed using the chi-square test or Fisher’s exact test, as appropriate. A p-value < 0.05 was considered statistically significant.

Ethical considerations

This study was approved by the institutional review board of Tokushukai Group Joint Ethics Review Committee (approval TGE02991-016), and the requirement for informed consent was waived due to the retrospective nature of the study.

## Results

Patient characteristics

A total of 86 patients (100%) were included in this study: 41 (47.7%) in the PICC group and 45 (52.3%) in the CVC group. There were no significant differences between the groups in baseline characteristics. The mean age was 73.1 ± 9.5 years in the PICC group and 72.7 ± 11.2 years in the CVC group (p = 0.830). The proportion of male patients was 68.3% (n = 28) in the PICC group and 60.0% (n = 27) in the CVC group (p = 0.565). Body mass index (BMI) was comparable between the groups (22.8 ± 3.7 vs. 21.3 ± 3.2 kg/m², p = 0.053) (Table 1).

**Table 1:** Patient characteristics Baseline demographic characteristics of patients undergoing general anesthesia with either peripherally inserted central catheter (PICC) placement or conventional central venous catheter (CVC) placement. Continuous variables are expressed as mean ± standard deviation (SD) and were compared using the Student’s t-test. Categorical variables were analyzed using the chi-square test. BMI, body mass index.

Variable	PICC (n=41)	CVC (n=45)	p value
Age (years), mean ± SD	73.1 ± 9.5	72.7 ± 11.2	0.830
Male, n (%)	28 (68.3%)	27 (60.0%)	0.565
BMI (kg/m²), mean ± SD	22.8 ± 3.7	21.3 ± 3.2	0.053

Procedural outcomes

All catheter placements were successfully completed in both groups (100% success rate).

**Figure 1:**
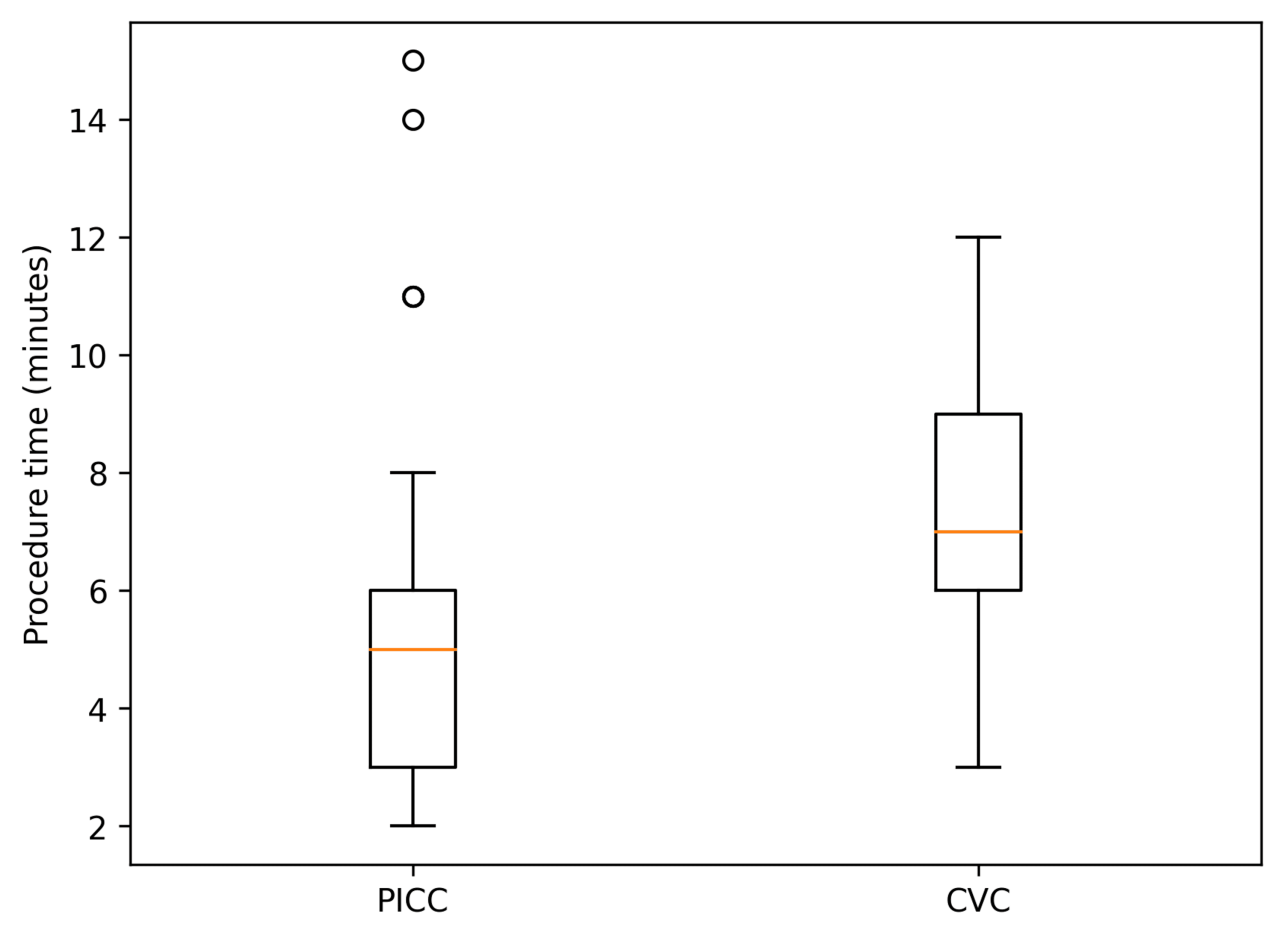
Comparison of procedure time between PICC and CVC groups Box-and-whisker plots showing procedure time (minutes) for peripherally inserted central catheter (PICC) placement and conventional central venous catheter (CVC) placement. The box represents the interquartile range (IQR), the horizontal line within the box indicates the median, and the whiskers represent the minimum and maximum values. Procedure time was significantly shorter in the PICC group (p = 0.013).

The procedure time was significantly shorter in the PICC group than in the CVC group (5.5 ± 3.1 vs. 7.0 ± 2.3 minutes, p = 0.013) (Figure 1).

The duration of catheter use was significantly longer in the PICC group (7.1 ± 3.1 days) compared with the CVC group (3.3 ± 1.7 days) (p < 0.001) (Table 2). Surgery duration (incision-to-closure time) tended to be longer in the CVC group than in the PICC group (527.9 ± 197.6 vs. 461.5 ± 144.8 min; p = 0.077) (Table 2).

**Table 2:** Comparison of procedural variables between the PICC and CVC groups Comparison of procedural variables between the peripherally inserted central catheter (PICC) and conventional central venous catheter (CVC) groups. Continuous variables are presented as mean ± SD and were compared using the Student’s t-test. Successful placement rates are expressed as percentages. Surgery duration was defined as incision-to-closure time.

Variable	PICC (n=41)	CVC (n=45)	p value
Procedure time (min), mean ± SD	5.5 ± 3.1	7.0 ± 2.3	0.013
Catheter duration (days), mean ± SD	7.1 ± 3.1	3.3 ± 1.7	<0.001
Successful placement, n (%)	41 (100%)	45 (100%)	–
Surgery duration, min (mean ± SD)	461.5 ± 144.8	527.9 ± 197.6	0.077

Catheter-related complications

CRBSI occurred in one patient (2.4%) in the PICC group and in four patients (8.9%) in the CVC group, with no statistically significant difference between groups (p = 0.363) (Table 3). In addition, one case of catheter occlusion with intraluminal thrombus was observed in the PICC group (1/41, 2.4%), whereas no such events were documented in the CVC group (0/45, 0%) (Table 3).

**Table 3:** Catheter-related complications Incidence of catheter-related complications in the peripherally inserted central catheter (PICC) and conventional central venous catheter (CVC) groups, including catheter-related bloodstream infection (CRBSI) and catheter occlusion with intraluminal thrombus. Categorical variables were analyzed using Fisher’s exact test due to the small number of events.

Variable	PICC (n=41)	CVC (n=45)	p value
Infection (CRBSI), n (%)	1 (2.4%)	4 (8.9%)	0.363
Catheter occlusion with intraluminal thrombus, n (%)	1 (2.4%)	0 (0.0%)	0.477

The distribution of surgical diagnoses was comparable between the two groups (p = 0.566). Hepatobiliary-pancreatic surgery accounted for the majority of cases in both groups (65.9%, n = 27 in the PICC group and 64.4%, n = 29 in the CVC group), followed by upper gastrointestinal surgery (19.5%, n = 8 vs. 15.6%, n = 7) and lower gastrointestinal surgery (14.6%, n = 6 vs. 15.6%, n = 7). Only two cases (4.4%, n = 2) categorized as “Other” were included in the CVC group (Table 4).

**Table 4:** Surgical diagnosis classification Distribution of surgical diagnosis categories in the peripherally inserted central catheter (PICC) and conventional central venous catheter (CVC) groups. Diagnoses were categorized into upper gastrointestinal, lower gastrointestinal, hepatobiliary–pancreatic, and other surgical procedures. Categorical variables were compared using the chi-square test.

Diagnosis Category	PICC (n=41)	CVC (n=45)	p value
Upper GI	8 (19.5%)	7 (15.6%)	
Lower GI	6 (14.6%)	7 (15.6%)	
Hepatobiliary-Pancreatic	27 (65.9%)	29 (64.4%)	
Other	0 (0%)	2 (4.4%)	0.566

## Discussion

In this retrospective single-center comparative cohort study, ultrasound-guided PICC placement during general anesthesia was associated with a shorter procedure time and a longer catheter duration than ultrasound-guided CVC placement. Catheter-related complications were infrequent in both groups; however, due to the non-randomized design and low event counts, definitive conclusions regarding comparative safety cannot be drawn. These findings support the feasibility of incorporating nurse-performed ultrasound-guided PICC placement into a structured perioperative vascular access workflow and suggest that protocolized task allocation may improve procedural efficiency. Prospective multicenter studies incorporating direct operating room workflow metrics and catheter-day-based complication rates are warranted.

Procedural efficiency and workflow design

The procedure time was significantly shorter in the PICC group. In our institutional workflow, PICC placement was performed by trained NPs under ultrasound guidance during general anesthesia. This protocolized and role-defined approach may have contributed to procedural efficiency. The use of specialized vascular access specialist teams (VAST) has been shown in previous systematic reviews to improve efficiency and reduce the overall incidence of complications [11].

Importantly, the observed reduction in procedure time should not be interpreted as intrinsic technical superiority of PICC over CVC. Conventional CVC placement has well-established clinical utility but is associated with mechanical risks, including arterial puncture and pneumothorax, particularly during peri-induction instability [1,3,4]. The benefits of ultrasound guidance in reducing insertion-related complications have been well documented [6]. In our study, both groups utilized ultrasound guidance, which may explain the low complication rate observed. The standardization of ultrasound-guided techniques has become a global gold standard, ensuring high success rates regardless of the operator's profession [6,12]. Rather than device superiority, the time difference likely reflects differences in task allocation and operating room workflow. Conventional CVC placement is typically performed by anesthesiologists while simultaneously managing airway and hemodynamic control. In contrast, nurse-led PICC placement allowed for parallel task execution within a coordinated perioperative team structure. From a systems perspective, structured task allocation has been recognized as an important strategy for optimizing procedural efficiency in high-acuity environments [8].

For patients who definitively require central venous access, preoperative PICC placement outside the operating room (e.g., in the holding area or on the ward) may further improve perioperative efficiency by shifting line insertion away from anesthesia-controlled time and minimizing task stacking during induction. This parallel workflow can allow anesthesiologists to prioritize airway management and hemodynamic stabilization while preserving operating room throughput and supporting seamless postoperative vascular access continuity. Implementation, however, requires a reliable vascular access team, clear patient selection criteria, and institutional protocols to avoid overuse. Potential barriers include resource constraints, scheduling/coordination challenges, concerns regarding thrombotic or occlusive events, and variability in scope-of-practice regulations across institutions.

Vascular access strategy and volume management

In our practice, PICC was not utilized as an isolated access device. When rapid infusion capability was anticipated, a large-bore peripheral intravenous line was secured in parallel. Therefore, PICC placement formed part of a comprehensive vascular access strategy rather than replacing conventional central venous access in all respects. Although PICCs offer advantages such as reduced insertion-related mechanical complications, their smaller internal diameter compared with conventional CVCs may limit their suitability for high-flow resuscitation. The present study did not directly evaluate flow performance or massive transfusion scenarios. However, international recommendations suggest that for major surgery, the choice of vascular access should be balanced between the need for high-flow resuscitation and the risk of mechanical complications associated with CVCs [13]. Accordingly, our findings should be interpreted within the context of a structured perioperative access plan that included adjunctive peripheral volume lines when clinically indicated.

Catheter duration and perioperative continuity

The catheter duration was significantly longer in the PICC group. This likely reflects anticipated postoperative management needs, as PICCs are frequently selected when extended vascular access is expected. The longer dwell time of PICCs facilitates a smoother transition from the operating room to the intensive care unit or general ward, providing reliable access for postoperative parenteral nutrition or intravenous medications [14]. The longer usable duration may contribute to reduced need for early catheter replacement and facilitate perioperative continuity of care. However, because catheter selection was not randomized, this difference may partially reflect clinical decision-making rather than device-specific characteristics.

Safety considerations

The incidence of catheter-related infection did not significantly differ between groups. Although numerically lower in the PICC group, the number of infectious events was small, limiting statistical power. Central line-associated bloodstream infection remains a major concern in perioperative and critical care settings. Large-scale observational data have indicated that while PICCs are not immune to infection, their infection rates are often comparable to or lower than those of CVCs when managed under strict sterile protocols [15,16]. Implementation of standardized insertion protocols and adherence to infection prevention bundles have been shown to substantially reduce infection risk [17]. In our institution, standardized ultrasound-guided insertion techniques and perioperative sterile protocols were applied to both groups, which may have contributed to the low observed infection rate. These results suggest that ultrasound-guided PICC placement by trained NPs can be performed safely under controlled operative conditions. Nevertheless, definitive conclusions regarding comparative infection risk cannot be drawn from the present dataset.

Implications for nurse-led task sharing

The integration of NPs into perioperative vascular access management represents an evolving collaborative practice model. In environments facing increasing procedural demand and workforce constraints, clearly defined task sharing has been proposed as a strategy to improve efficiency while maintaining safety [8]. Globally, advanced practice nurses have successfully assumed procedural roles, with studies showing that nurse-led vascular access services provide outcomes non-inferior to physician-led services [10,12]. Our findings indicate that nurse-led PICC placement can be incorporated into perioperative workflows as part of a structured vascular access strategy without compromising short-term safety outcomes. Importantly, this model does not replace physician expertise but complements anesthesiologist-led care within a coordinated perioperative team.

Future prospective studies incorporating direct operating room workflow metrics (e.g., induction-to-incision time and anesthesia-controlled time) and appropriate adjustment for confounding are needed to confirm these system-level effects.

Limitations

This study has several limitations. First, it was a single-center retrospective observational study with non-random device selection driven by institutional workflow and staffing (weekday daytime NP availability vs on-call/NP unavailable), which introduces confounding by indication and limits causal inference. Second, catheter dwell time was longer in the PICC group, likely reflecting anticipated postoperative needs and clinical selection; therefore, comparisons of catheter-related complications should be interpreted cautiously. Third, the number of infectious and occlusive events was low, and the study was underpowered to draw definitive conclusions regarding comparative safety. Fourth, complication capture was limited by the retrospective design. Routine imaging screening for venous thrombosis was not performed; thus, the true incidence of catheter-associated venous thrombosis could not be determined, and we reported only clinically apparent catheter occlusion with intraluminal thrombus identified upon removal. In addition, catheter-day-based infection rates were not available in this dataset. Finally, generalizability may be limited because the findings reflect a Japanese single-institution perioperative workflow and a nationally certified NP training model; results may differ in settings with different staffing structures, scope-of-practice regulations, and vascular access protocols.

## Conclusions

In conclusion, ultrasound-guided PICC placement during general anesthesia was associated with shorter procedure time and longer catheter dwell duration than ultrasound-guided CVC placement in this single-center retrospective cohort. Catheter-related complications were infrequent; however, due to the non-randomized design, potential confounding by indication, and low event counts, definitive conclusions regarding comparative safety cannot be drawn. Our findings support the feasibility of nurse-performed ultrasound-guided PICC placement within a structured perioperative workflow and suggest that protocolized task allocation may improve procedural efficiency. Prospective multicenter studies incorporating direct operating room workflow metrics and catheter-day-based outcomes are warranted.
